# 

**Published:** 1847-03

**Authors:** 


					Vol. VII.
MUCH, 1847.
H
So. I.
THE
AMERICAN
???l I inn II
J
'"" '~   ' " '? - ?
OF
^ ~ienc J
mk f?s3?
QUARTERLY.
??     j " ii _
PUBLISHED UNDER THE AUI*(CSS 0* *H*
^mrrfcan SocCets of mtntul &ux&ton*
i!$?
CHAPIN A. HARRIS, M. D., D./B.
AMOS WESTCOTT, M. D., D. D. S.
EDWIN J. DUNNING.
CORRESPONDENT FOR ENGLAND
rsON, D. D. S.,7 Gower-st. Bedford Square,
BALTIMORE:
ARMSTRONG & BERRT,;
PHILADELPHIA:
W. WOODS, PRINTtE.
annum, payable in adTaMe.t
This Periodical weighs 9 ounces.

				

